# Early and late mortality after malaria in young children in Papua, Indonesia

**DOI:** 10.1186/s12879-019-4497-y

**Published:** 2019-10-30

**Authors:** Dewi Patriani, Eggi Arguni, Enny Kenangalem, Saber Dini, Paulus Sugiarto, Afdhal Hasanuddin, Daniel Adrian Lampah, Nicholas M. Douglas, Nicholas M. Anstey, Julie Anne Simpson, Ric N. Price, Jeanne Rini Poespoprodjo

**Affiliations:** 1 Department of Child Health, Faculty of Medicine, Public Health and Nursing, Universitas Gadjah Mada, Jl. Kesehatan no.1, Sekip, Yogyakarta, 55284 Indonesia; 2 Timika Malaria Research Programme, Papuan Health and Community Development Foundation, Jl. SP2-SP5, RSMM Area, Timika, Papua 99910 Indonesia; 3 Mimika District Hospital, Jl. Yos Sudarso, Timika, Papua 99910 Indonesia; 4 Centre for Epidemiology and Biostatistics, Melbourne School of Population and Global Health, 207 Bouverie Street, The University of Melbourne, Melbourne, Victoria 3010 Australia; 5 Mitra Masyarakat Hospital, Jl. SP2-SP5-Charitas, Timika, 99910 Indonesia; 6 Global and Tropical Health Division, Menzies School of Health Research and Charles Darwin University, PO Box 41096, Casuarina, Darwin, NT 0811 Australia; 7 Division of Medicine, Royal Darwin Hospital, Darwin, NT 0810 Australia; 8 Centre for Tropical Medicine and Global Health, Nuffield Department of Clinical Medicine, University of Oxford, OX37LJ, Oxford, United Kingdom; 9 Mahidol-Oxford Tropical Medicine Research Unit, Faculty of Tropical Medicine, Mahidol University, Bangkok, Thailand

**Keywords:** *P. falciparum*, *P. vivax*, Infants and children under-fives, Mortality

## Abstract

**Background:**

In southern Papua, Indonesia, malaria is highly prevalent in young children and is a significant cause of morbidity and early mortality. The association between malaria and delayed mortality is unknown.

**Methods:**

Routinely-collected hospital surveillance data from southern Papua, Indonesia, were used to assess the risk of recurrent malaria and mortality within 12 months of an initial presentation with malaria in all children younger than 5 years old attending the local hospital. Analysis was primarily by Kaplan Meier and Cox regression methods.

**Results:**

In total 15,716 children presenting with malaria between April 2004 and December 2013 were included in the analysis; 6184 (39.3%) with *Plasmodium falciparum,* 7499 (47.7%) with *P. vivax*, 203 (1.3%) with *P. malariae,* 3 with *P. ovale* and 1827 (11.6%) with mixed infections*.* Within 1 year, 48.4% (7620/15,716) of children represented a total of 16,957 times with malaria (range 1 to 11 episodes), with the incidence of malaria being greater in patients initially presenting with *P. vivax* infection (1334 [95%CI 1307–1361] per 1000 patient years) compared to those with *P. falciparum* infection (920 [896–944]). In total 266 (1.7%) children died within 1 year of their initial presentation, 129 (48.5%) within 30 days and 137 (51.5%) between 31 and 365 days. There was no significant difference in the mortality risk in patients infected with *P. vivax* versus *P. falciparum* either before 30 days (Hazard Ratio (HR) 1.02 [0.69,1.49]) or between 31 and 365 days (HR = 1.30 [0.90,1.88]). Children who died had a greater incidence of malaria, 2280 [95%CI 1946–2671] per 1000 patient years preceding their death, compared to 1141 [95%CI 1124–1158] per 1000 patient years in those surviving.

**Conclusions:**

Children under-5 years old with *P. vivax* malaria, are at significant risk of multiple representations with malaria and of dying within 1 year of their initial presentation. Preventing recurrent malaria must be a public health priority in this vulnerable population.

## Background

Children under 5 years of age, living in malaria endemic areas are particularly vulnerable to malaria, its severe manifestations and malaria attributable mortality [1]. In Papua Province, eastern Indonesia, recurrent episodes of malaria occur frequently in early life and are associated with significant morbidity and mortality [2, 3]. The severe manifestations of *Plasmodium falciparum* malaria are well recognized, and there is now good evidence of early morbidity and mortality associated with *Plasmodium vivax* infection in some endemic regions [3, 4]. Pauci-immune young children are at risk of recurrent episodes of malaria, and in Asia this is particularly apparent following *P. vivax* infection*.*

Malaria control and elimination efforts are more challenging for *P. vivax* than *P. falciparum* and consequently in many co-endemic settings, there has been a rise in the relative proportion of malaria attributable to *P. vivax*. Furthermore without radical cure of the dormant liver stages of *P. vivax*, recurrent parasitaemia occurs every 3–4 weeks in equatorial regions [5] resulting in a cumulative risk of severe anaemia [6].

The aetiology of *P. vivax* mortality remains controversial, however malnutrition, severe anaemia and bacterial sepsis are potentially important contributing factors [7, 8]. The long-term impact of recurrent malaria in young children is poorly described. This study aimed to quantify and compare the early and late mortality of children less than 5 years of age following presentation with *P. vivax* and/or *P. falciparum* infection in Papua, Indonesia.

## Methods

### Study site

Timika is located in southern Papua, the most easterly province of Indonesia. A local census in 2012, recorded the population of Mimika district to be 202,350 with almost 90% of the population located in Timika town and surrounding villages serviced by the Mitra Masyarakat hospital (RSMM). The area has fragmented forest with a climate that varies little throughout the year (unpublished data). Malaria is restricted to lowland areas where transmission is unstable. The annual incidence of clinical malaria during the study period declined from 889 per 1000 population in 2004 to 522 per 1000 in 2013 and over the same period the proportion of infections due to *P. vivax* rose from 44 to 53% [9]. Local *P. vivax* strains have a typical relapse interval of 3–4 weeks and are highly resistant to chloroquine [10, 11]. Approximately half of the population own insecticide treated bednets [12].

Rumah Sakit Mitra Masyarakat (RSMM) is the largest health care facility in Timika. During the study period, RSMM treated between 40 and 60% of patients diagnosed with malaria in the district [12]. A second hospital opened in December 2008, but did not begin seeing substantial numbers of malaria patients until 2010. From 2010 to 2013, RSMM received an estimated 80% of malaria patients attending either of the two hospitals [9]. RSMM offers free medical care for most patients of lowland or highland Papuan ethnicity, who make up 93% of attendees, and as such is the preferred source of medical care for inpatient, outpatient and antenatal encounters [12]. A community household survey of treatment seeking behavior in 2005, estimated that more than 80% of children under 5 who had died in the preceding year had done so at the RSMM hospital [13].

### Study population

The local population is made up of ethnic groups that can be divided broadly into highland and lowland Papuans, with a migrant non-Papuan population originating mostly from low or non-malaria endemic regions. Diarrhea, lower respiratory tract infections and malaria are the commonest causes of morbidity and mortality in young children. Excluding neonatal deaths, the under-five mortality rate in babies born at RSMM between 2004 and 2013 was 17 per 1000 live births (unpublished data). Insecticide treated bednets have been widely distributed since 2004 and are used by approximately half of the population [12].

### Study design

Data were gathered prospectively from routine documentation of patients presenting to the RSMM hospital. No patient data were gathered on events occurring at other healthcare facilities or outlets. Between April 2004 and December 2013 patients presenting to RSMM had clinical, laboratory and pharmacy data recorded in a Q-Pro™ database and could be identified by a unique hospital record number. Data recorded included date of presentation and discharge, age, sex, ethnicity, clinical diagnoses (ICD 10) assigned by the attending physician, full blood count parameters and prescription details. The analysis included all children aged 5 years or under at the time of their first presentation (either as an inpatient or outpatient) to hospital with malaria. All subsequent visits to hospital were recorded, including re-presentation with and without malaria. Babies in the first month of life were excluded to avoid confounding from perinatal mortality and congenital malaria [14, 15]. Hospital protocol dictates that any patient presenting with fever, a history of fever or severe illness should be checked for malaria by microscopy using Giemsa stained thick blood smears. Review and quality assurance demonstrated that this protocol was well adhered to with microscopy accuracy of 90% compared to expert review [3]. Prior to 2006, the first line local treatment for uncomplicated malaria was chloroquine plus sulphadoxine-pyrimethamine for *P. falciparum* and chloroquine for *P. vivax*, although in the RSMM hospital most patients were treated with a 7 day oral regimen of quinine. Patients with *P. vivax* were also offered a 14 day course of low-dose primaquine (3.5 mg/kg total dose). In view of the poor efficacy of these regimens, the standard protocol was changed in March 2006 to dihydroartemisinin-piperaquine (DHP) for all *Plasmodium* species plus 14 days primaquine in those with *P. vivax* infection [16]. At the same time the recommended treatment for severe malaria was changed from intravenous quinine to intravenous artesunate [17].

### Definitions

Infants were defined as children aged less than 1 year old and young children as those aged 1 to less than 5 years old. Children under 5 years old include both infants and young children. A diagnosis of malaria was only made following confirmation of parasitaemia by blood film microscopy. Mortality was defined as death during admission due to any cause and categorized as early (within 30 days) or late (from 31 to 365 days of the first hospital presentation with malaria). Severe anaemia was defined as a haemoglobin concentration below 7 g/dl. Malnutrition was assessed by the attending physician and defined as weight for age or height/length for weight z scores below -3SD of standard reference at that time [18, 19].

### Statistical analysis

Data were analysed using SPSS version 20.0 for windows software (IBM SPSS Statistics) and Stata version 14.2 (StataCorp, College Station, TX, US). Clinical, laboratory and pharmacy data were concatenated such that multiple presentations with malaria within 14 days of the initial presentation were grouped as a single “episode”, the rationale being that recurrent parasitemia due to reinfection or relapse is highly unlikely within this period [20]. Kaplan-Meier survival curves for the risk of re-presentation to hospital with malaria and the risk of any hospital presentation terminating in death were plotted stratified by the baseline characteristics sex, ethnicity (non-Papuan, Highland Papuan, Lowland Papuan), age group, year of initial presentation, presence or absence of *Plasmodium* co-infection and initial admission status (outpatient or inpatient). Cox proportional hazards regression models were used to identify risk factors for recurrent malaria and early and late mortality. The proportional hazards assumption was assessed by comparing visually the log (cumulative hazard) by time of follow-up curves for each covariable category and subsequently by fitting and comparing models with and without time of follow-up interaction terms. Univariable analyses were performed for each of the following variables: *Plasmodium* species, sex, self-reported ethnicity (non-Papuan, Highland Papuan, Lowland Papuan), age group (< 1 year and 1 to < 5 years), inpatient status, presence of severe anaemia (Hb < 7 g/dl or < 5 g/dl) and malnutrition. All of these factors were included in multivariable models. In view of the change of treatment policy, antimalarial treatment regimens were collinear with the year of the study, thus all multivariable models were stratified by year. For late mortality, we also performed subgroup analyses by whether the child was an outpatient for the initial malaria episode or admitted to hospital for management of the infection.

In survival analysis and the calculation of incidence rates, the period of observation was defined as the time from first presentation with malaria until 12 months, the time of recorded death or the end of the study period (31st December 2013), whichever occurred earliest. Population attributable fractions (PAF) were calculated using STATA 14.2 to calculate PAFs as described previously [21].

### Ethical approval

Ethical approval for this study was obtained from the Medical and Health Research Ethics Committee, Faculty of Medicine, Gadjah Mada University, Yogyakarta, Indonesia (KE/FK/544/EC), Menzies School of Health Research, Darwin, Australia (HREC 10.1397).

## Results

**Fig. 1:**
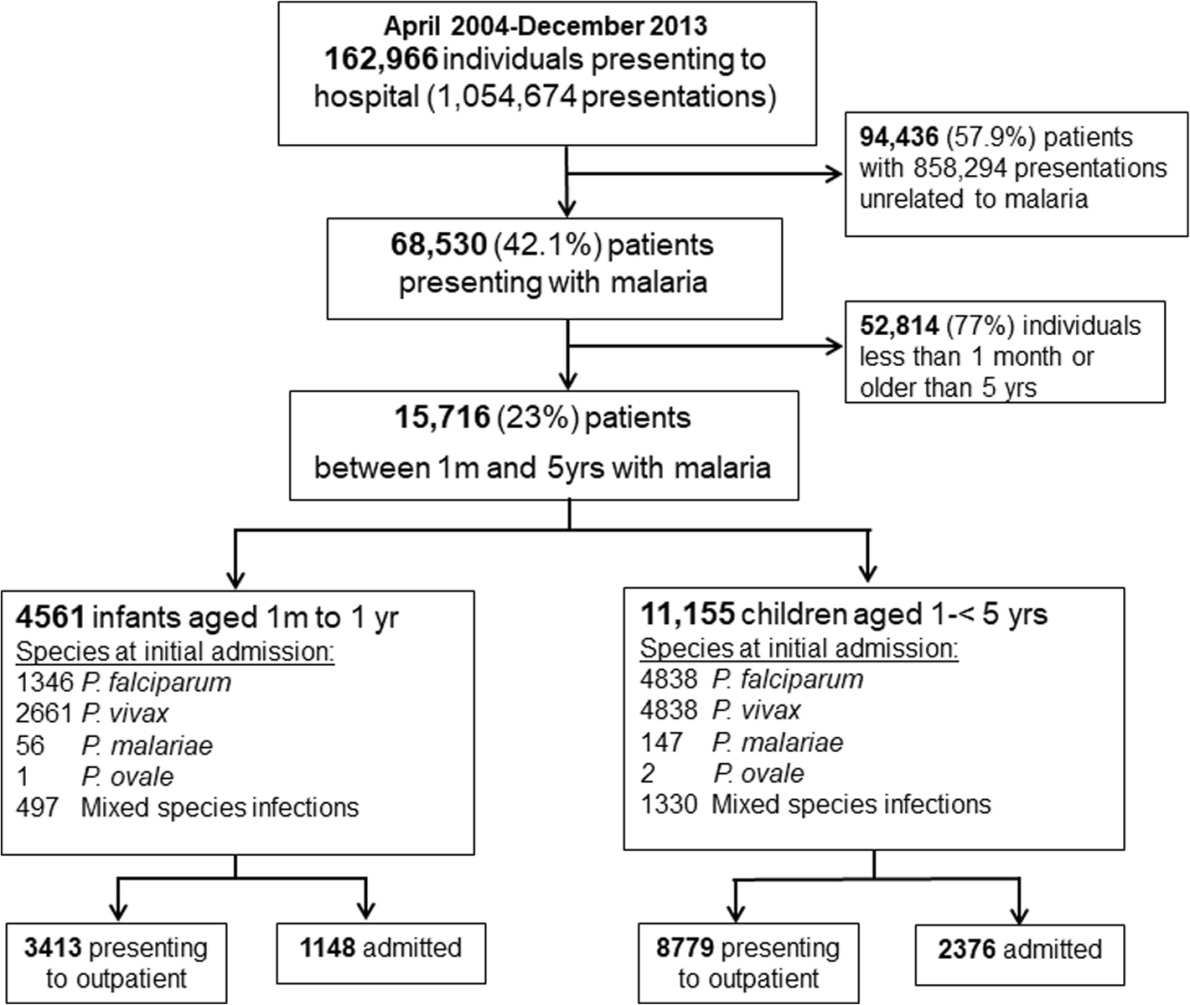
Study Profile

Between April 2004 and December 2013, 1,054,674 clinical presentations to RSMM were recorded, of which 196,380 (18.6%) episodes in 68,530 patients included a diagnosis of malaria. A total of 15,716 children were aged between 1 month and 5 years old at their first presentation to hospital with malaria, of whom 6184 (39.3%) had *P. falciparum* malaria, 7499 (47.7%) had *P. vivax*, 203 (1.3%) had *P. malariae*, 3 (0.02%) had *P. ovale* and 1827 (11.6%) had mixed infections. Overall 12,192 (77.6%) were treated as outpatients and 3524 (22.4%) required admission for inpatient care. The study profile is presented in Fig. 1 and the baseline characteristics in Table 1.

**Table 1:** Baseline characteristics of children under 5 years old at their first presentation to hospital with malaria

Characteristics	Total *n* = 15,716	Inpatients *n* = 3524	Outpatients *n* = 12,192
Age, years (%)			
Median [Range]	1.7 [0.1, 5.0]	1.5 [0.1, 5.0]	1.8 [0.1, 5.0]
1–5 years	11,155 (71.0)	2376 (67.4)	8779 (72.0)
< 1 year	4561 (29.0)	1148 (32.6)	3413 (28.0)
Sex, *n* (%)			
Females	7462 (47.5)	1656 (47.0)	5806 (47.6)
Male	8254 (52.5)	1868 (53.0)	6386 (52.4)
Ethnic group^a^, *n* (%)			
Non Papuan	1100 (7.0)	324 (9.2)	776 (6.4)
Highland Papuan	11,714 (74.5)	2516 (71.4)	9198 (75.4)
Lowland Papuan	2889 (18.4)	679 (19.3)	2210 (18.1)
Haemoglobin concentration, *n* (%)	*n* = 8687 (55.3%)	*n* = 3201 (90.8%)	*n* = 5486 (45.0%)
Mean Haemoglobin [SD] g/dl	8.1 [2.4]	7.3 [2.8]	8.6 [2.0]
Hb < 7 g/dl	2674 (30.8)	1441 (40.9)	1233 (10.1)
Hb < 5 g/dl	974 (11.2)	814 (23.1)	160 (1.3)
Malnourished, *n* (%)			
Normal	15,497 (98.6)	3368 (95.6)	12,129 (99.5)
Undernourished	219 (1.4)	156 (4.4)	63 (0.5)
Species of malaria, *n* (%)			
*P. falciparum*	6184 (39.3)	1818 (51.6)	4366 (35.8)
*P. vivax*	7499 (47.7)	1146 (32.5)	6353 (52.1)
*P. ovale*	3 (0.02)	0 (0.0)	3 (0.0)
*P. malariae*	203 (1.3)	25 (0.7)	178 (1.5)
Mixed Infections	1827 (11.6)	535 (15.2)	1292 (10.6)

*SD* standard deviation; ^a^Data missing for 13 children

At the initial presentation, 25.2% (1148/4561) of infants and 21.3% (2376/11,155) of young children required hospitalization for treatment of their malaria. Children presenting with *P. falciparum* infection were more likely to require inpatient treatment compared to those with *P. vivax* (Odds Ratio (OR) = 2.31 [95%CI 2.12–2.51], *p* < 0.001). Haemoglobin concentration was recorded during the hospital presentation in 8687 (55.3%) of children and the mean haemoglobin was 8.12 g/dl [SD = 2.4]. Severe anaemia was present in 30.8% (2674/8687) of cases (Hb < 7 g/dl).

The first episode of malaria could be matched with pharmacy records in 92.1% (14,472/15,716) of cases. Prior to March 2006, 69.2% (2341/3385) received oral treatment with quinine or chloroquine and 23.2% (784/3385) were treated with intravenous quinine with or without an oral regimen. Following the change in policy in March 2006, 96.6% (10,708/11,087) received oral treatment with an artemisinin-based combination therapy (ACT) and 19.3% (2136/11,087) were treated with intravenous artesunate with or without an oral regimen. In total 52.6% (7693/14,627) of children were also prescribed primaquine (PQ), of whom 23.5% (1810) were prescribed a single dose of PQ and 70.3% (5405) were prescribed 14 day regimens; for 478 (6.2%) children, the dose of PQ could not be defined.

### Risk factors for recurrent malaria

**Fig. 2:**
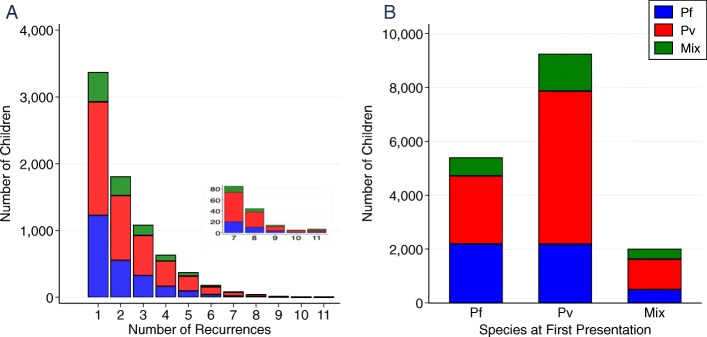
(**a**) Histogram showing the distribution of the number of recurrences of malaria stratified by *Plasmodium* species at recurrence – the first presentation is excluded (**b**) Total number of recurrences stratified by *Plasmodium* species at initial presentation and at recurrence

Within 12 months of their first presentation 7620 (48.4%) of children had at least one re-presentation with malaria (total number of re-presentations = 16,958, range 1 to 11); Fig. 2a. In infants, 63.9% (4144/6481) of all re-presentations with malaria were due to *P. vivax*, but this fell to 50.4% (5280/10,477) in young children; *p* < 0.0001. The incidence of malaria within 1 year was 1334 [95%CI 1307–1361] per 1000 person-years (py) in patients initially presenting with *P. vivax* compared to 920 [896–944] per 1000 py for those initially presenting with *P. falciparum*, Rate ratio 1.45 [95%CI 1.40–1.50], *p* < 0.001; Table 2.

**Table 2:** Malaria Incidence Rate within 1 year of initial presentation (per 1000 patient years (95% CI))

Outcome 12 months follow-up	Species at initial presentation			
	Any Malaria	*P. falciparum*	*P. vivax*	Mixed Infection
Any malaria	1146 (1129, 1164)	920 (896, 944)	1334 (1307, 1361)	1207 (1155, 1260)
*P. falciparum*	334 (325, 343)	370 (355, 386)	314 (301, 327)	297 (272, 324)
*P. vivax*	637 (624, 650)	425 (409, 442)	815 (794, 837)	673 (635, 713)
Mixed Infections	166 (160, 173)	115 (106, 123)	197 (187, 208)	227 (206, 251)
Inpatient admission with malaria	411 (401, 422)	361 (346, 376)	457 (441, 473)	415 (385, 447)
Outpatient presentation with malaria	3386 (3356, 3416)	2875 (2832, 2919)	3859 (3813, 3906)	3289 (3203, 3377)

At 12 months, the cumulative risk of any recurrent malaria was 58.9% [95%CI 57.4–60.3] in infants compared to 46.7% [95% CI 45.8–47.7] in young children; Hazard Ratio (HR) = 1.41 [95% CI: 1.34–1.47], *p* < 0.001 (Table 2).

**Fig. 3:**
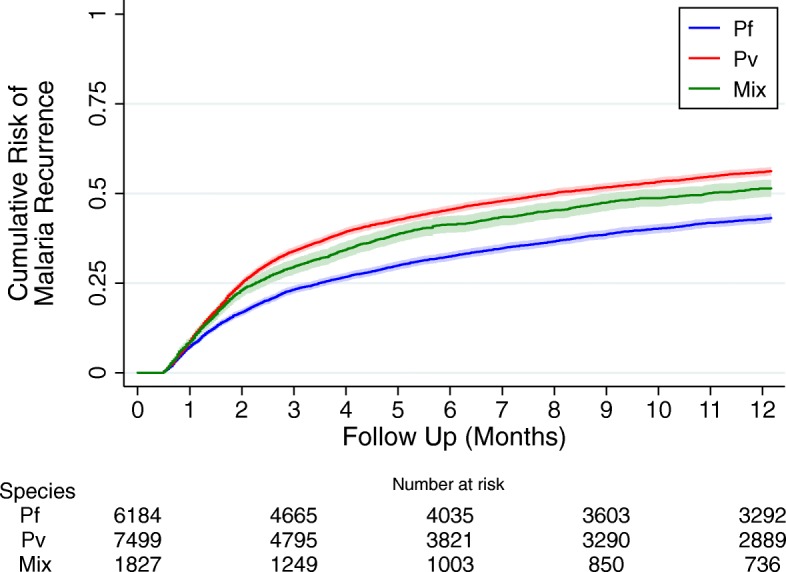
Risk of representation with malaria within 1 year after initial presentation. Figure stratified by the species at first presentation

Children initially infected with *P. vivax* were at greater risk of recurrent malaria compared to those initially infected with *P. falciparum* (HR = 1.48 [95%CI: 1.41–1.55]), *p* < 0.001); Figs. 2b, 3 and Table 3. Other univariable risk factors for re-presentation with malaria were highland ethnicity (HR = 3.70 [95% CI: 3.23–4.24]; *p* < 0.001), admission to hospital (HR = 0.73 [95%CI: 0.69–0.77]; *p* < 0.001), severe anaemia (HR = 0.89 [95%CI: 0.83–0.95]; *p* < 0.001) and malnutrition (HR = 0.64 [95% CI: 0.51–0.82], *p* < 0.001). In multivariable analysis, age, ethnicity, outpatient status, species of initial infection absence of malnutrition and absence of severe anaemia remained significant risk factors for representation with malaria; Table 3.

**Table 3:** Risk factors for malaria re-presentation to hospital within 1 year of initial infection

				Anaemia included in the model (*n* = 8680)			Anaemia excluded from the model (*n* = 15,703)		
Characteristics	Risk (num. of events/total cases)	Univariable Analysis Hazard ratio (95% CI)	*p* value	Multivariable Analysis Adjusted Hazard Ratio (95% CI)	*p* value	PAF % (95% CI)	Multivariable Analysis Adjusted Hazard Ratio (95% CI)	*p* value	PAF % (95% CI)
Age Group									
> =1 to <=5	45.2 (5044/11155)	Reference		Reference			Reference		
< 1	56.5 (2576/4561)	1.41 (1.34, 1.47)	< 0.001	1.35 (1.26, 1.44)	< 0.001	10.34 (7.97,12.66)	1.32 (1.26, 1.38)	< 0.001	8.84 (7.22,10.44)
Gender									
Female	48.1 (3587/7462)	Reference		Reference			Reference		
Male	48.9 (4033/8254)	1.01 (0.97, 1.06)	0.562	1.04 (0.97, 1.10)	0.266	1.89 (−1.49, 5.15)	1.02 (0.98, 1.07)	0.39	1.04 (−1.35, 3.37)
Ethnicity^a^									
Non Papuan	19.4 (213/1100)	Reference		Reference			Reference		
Highland	55.2 (6466/11714)	3.70 (3.23, 4.24)	< 0.001	3.90 (3.21, 4.75)	< 0.001	65.34 (60.35,69.71)	3.48 (3.03, 3.99)	< 0.001	62.53 (58.68,66.02)
Lowland	32.5 (939/2889)	1.75 (1.51, 2.03)	< 0.001	1.90 (1.54, 2.35)	< 0.001	5.05 (3.66, 6.41)	1.71 (1.48, 1.99)	< 0.001	4.25 (3.22, 5.26)
Inpatient Status									
Outpatient	50.9 (6207/12192)	Reference		Reference			Reference		
Inpatient	40.1 (1413/3524)	0.73 (0.69, 0.77)	< 0.001	0.82 (0.76, 0.88)	< 0.001	−6.68 (−9.01,-4.40)	0.78 (0.74, 0.83)	< 0.001	−4.85 (−5.97,-3.75)
Anaemia‡									
Hb > = 7 g/dl	48.3 (2904/6013)	Reference		Reference			–	–	–
Hb < 7 g/dl	44.2 (1182/2674)	0.89 (0.83, 0.95)	< 0.001	0.89 (0.83, 0.96)	0.002	−3.29 (−5.40,-1.23)	–	–	–
Malnourished									
Normal	48.7 (7552/15497)	Reference		Reference			Reference		
Undernourished	31.1 (68/219)	0.64 (0.51, 0.82)	< 0.001	0.75 (0.58, 0.97)	0.026	−0.51 (−0.90,-0.12)	0.75 (0.59, 0.96)	0.021	−0.29 (−0.50,-0.07)
Species at presentation									
*P. falciparum*	42.0 (2597/6184)	Reference		Reference			Reference		
*P. vivax*	54.0 (4048/7499)	1.48 (1.41, 1.55)	< 0.001	1.24 (1.16, 1.33)	< 0.001	10.14 (6.82,13.34)	1.31 (1.24, 1.37)	< 0.001	13.11 (10.61,15.53)
*P. ovale*	66.7 (2/3)	1.99 (0.50, 7.97)	0.33	3.58 (0.89,14.40)	0.073	0.06 (−0.06, 0.18)	2.54 (0.63,10.18)	0.188	0.02 (− 0.03, 0.08)
*P. malariae*	37.4 (76/203)	0.85 (0.68, 1.07)	0.163	0.75 (0.54, 1.05)	0.096	−0.25 (− 0.51, 0.01)	0.82 (0.65, 1.03)	0.088	− 0.19 (− 0.40, 0.01)
Mix	49.1 (897/1827)	1.30 (1.20, 1.40)	< 0.001	1.20 (1.09, 1.33)	< 0.001	2.37 (1.02, 3.69)	1.19 (1.10, 1.29)	< 0.001	1.92 (1.02, 2.8)

All multivariable models were stratified by year. In view of the change in antimalarial treatment policy in 2006 and collinearity of year and treatment, oral and intravenous therapy received was not entered in the multivariable models. *PAF* population attributable fraction. ^a^ Data missing for 13 children

### Mortality

**Fig. 4:**
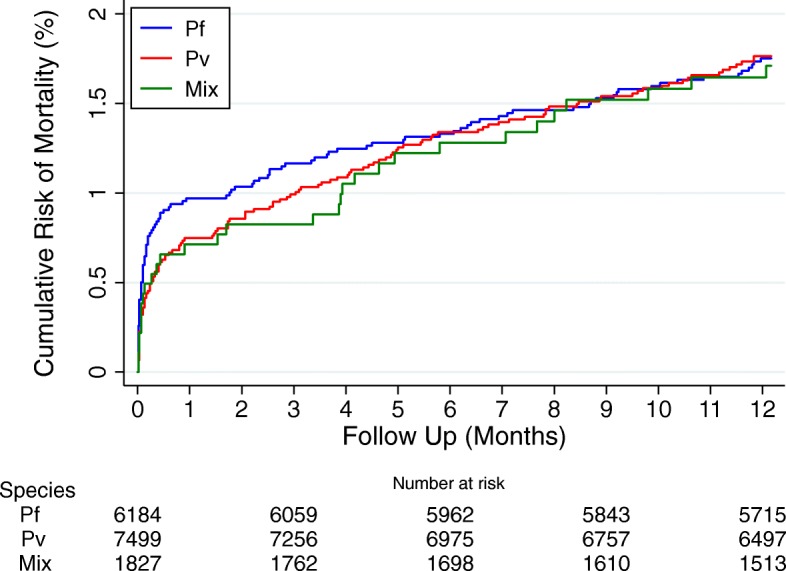
Risk of mortality within 1 year after initial presentation. Figure stratified by the species at first presentation

Of the 15,716 children presenting to the hospital, 266 children died within 12 months with a cumulative risk of 1.7% (95%CI: 1.5–1.9). Of these 266 deaths, 94 (35.3%) occurred within the first 7 days of the initial presentation, 35 (13.2%) between days 8 to 30, and 137 (51.5%) between days 31 and 365 (Fig. 4).

#### Early mortality

The risk of early mortality was non-significantly higher in infants (1.0% [95%CI: 0.8–1.3]) compared to young children (0.7% [95%CI: 0.6–0.9]; *p* = 0.095) and those presenting with *P. falciparum* infection (1.0% [95%CI, 0.7–1.2]) compared *P. vivax* infection (0.7% [95%CI, 0.6–1.0]; *p* = 0.156).

Early mortality was significantly more likely in undernourished children (HR = 19.65 [95%CI: 12.86–30.04]); *p* < 0.001), children initially requiring hospitalization (HR = 10.18 [95%CI, 6.85–15.12], *p* < 0.001) and those presenting with severe anaemia (HR = 2.03 [95%CI: 1.41–2.94], *p* < 0.001); Table 4. Compared to non-Papuans, highland ethnicity was associated with a lower risk of mortality (HR = 0.46 [95%CI 0.27–0.77], *p* = 0.003). In multivariable analysis, ethnicity, inpatient status, malnutrition and presenting with severe anaemia remained significant risk factors for early mortality; Table 4. Among undernourished children with malaria, the risk of dying within 30 days was 10.5% (9/86) in children infected with *P. falciparum* and 15.5% (15/97) in those infected with *P. vivax*; *p* = 0.437. In the 2664 (17.0%) children initially requiring admission to hospital and intravenous treatment with either quinine or artesunate, 3.4% (28/835) of those with *P. vivax* died within 30 days compared to 1.7% (24/1375) of those presenting with *P. falciparum* (HR = 1.93 [95%CI 1.12–3.34]; *p* = 0.018).

**Table 4:** Risk Factors for early mortality (within 30 days)

				Anaemia included in the model (*n* = 8680)			Anaemia excluded from the model (*n* = 15,703)		
Characteristics	Risk (Events/total cases)	Univariable Analysis Hazard ratio (95% CI)	*p* value	Multivariable Analysis Adjusted hazard ratio (95% CI)	*p* value	PAF % (95% CI)	Multivariable Analysis Adjusted hazard ratio (95% CI)	*p* value	PAF % (95% CI)
Age Group									
> =1 to <=5	0.7 (83/11155)	Reference		Reference			Reference		
< 1	1.0 (46/4561)	1.36 (0.95, 1.95)	0.097	1.08 (0.72, 1.61)	0.709	2.42 (−11.32,14.47)	1.24 (0.86, 1.80)	0.246	6.94 (−5.97,18.28)
Gender									
Female	0.8 (62/7462)	Reference		Reference			Reference		
Male	0.8 (67/8254)	0.98 (0.69, 1.38)	0.893	0.92 (0.64, 1.33)	0.662	−4.40 (−26.19,13.62)	0.96 (0.68, 1.36)	0.813	−2.21 (−22.31,14.58)
Ethnicity^a^									
Non Papuan	1.5 (17/1100)	Reference		Reference			Reference		
Highland	0.7 (83/11714)	0.46 (0.27, 0.77)	0.003	0.39 (0.23, 0.67)	< 0.001	−96.50 (−192.11,-32.18)	0.51 (0.30, 0.87)	0.013	−59.72 (−133.26,-9.36)
Lowland	1.0 (29/2889)	0.65 (0.35, 1.17)	0.152	0.45 (0.24, 0.85)	0.014	−25.96 (−53.50,-3.37)	0.60 (0.33, 1.10)	0.098	−14.67 (−36.25, 3.48)
Inpatient Status									
Outpatient	0.3 (33/12192)	Reference		Reference			Reference		
Inpatient	2.7 (96/ 3524)	10.18 (6.85, 15.12)	< 0.001	4.29 (2.69, 6.86)	< 0.001	60.02 (43.07,71.93)	8.22 (5.37,12.57)	< 0.001	66.56 (54.43,75.46)
Anaemia									
Hb > = 7 g/dl	1.0 (60/6013)	Reference		Reference			–	–	–
Hb < 7 g/dl	2.0 (54/2674)	2.03 (1.41, 2.94)	< 0.001	1.56 (1.05, 2.30)	0.027	17.49 (0.31,31.71)	–	–	–
Malnourished									
Normal	0.7 (102/15497)	Reference		Reference			Reference		
Undernourished	12.3 (27/ 219)	19.65 (12.86, 30.04)	< 0.001	9.19 (5.82,14.49)	< 0.001	22.38 (13.37,30.45)	8.88 (5.66,13.92)	< 0.001	19.93 (11.81,27.31)
Species at Presentation									
*P. falciparum*	1.0 (60/6184)	Reference		Reference			Reference		
*P. vivax*	0.7 (56/7499)	0.77 (0.53, 1.11)	0.157	1.05 (0.70, 1.57)	0.824	1.92 (−16.59,17.50)	1.02 (0.69, 1.49)	0.935	0.67 (− 16.96,15.64)
Mix	0.7 (13/1827)	0.73 (0.40, 1.34)	0.311	0.63 (0.32, 1.21)	0.166	−5.59 (−12.73, 1.10)	0.65 (0.36, 1.21)	0.175	−5.24 (− 12.19, 1.29)

All multivariable models were stratified by year. In view of the change in antimalarial treatment policy in 2006 and collinearity of year and treatment, oral and intravenous therapy received was not entered in the multivariable models. *PAF* population attributable fraction. ^a^ Data missing for 13 children

#### Late mortality

**Fig. 5:**
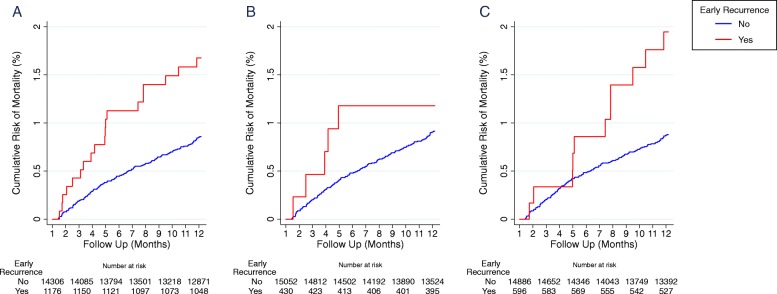
Risk of mortality stratified on the recurrence of malaria within 30 days of first presentation. **a** any parasitaemia, **b** *P. falciparum* parasitaemia and **c** *P. vivax* parasitaemia. Footnote: Red: parasitaemia within 30 days; Blue: no parasitaemia within 30 days

In total 137 (0.9%) children died between 31 and 365 days of their initial presentation. The risk of late mortality was 1.0% [95%CI 0.8–1.3] in children initially presenting with *P. vivax*, 0.8% [95%CI 0.6–1.0] in those initially presenting with *P. falciparum* and 1.0% [95%CI 0.6–1.6] in those with mixed infections; *p* = 0.34. The risk of late mortality was greater in infants than older children (HR = 3.11 [95%CI: 2.22–4.35], *p* < 0.001), children requiring admission to hospital compared to those initially managed as an outpatient (HR = 1.83 [95%CI: 1.28–2.60], *p* = 0.004), children who were undernourished (HR = 2.99 [95%CI: 1.22–7.3], *p* = 0.016) and those who were severely anaemic (HR = 1.83 [95%CI: 1.21–2.79]; *p* = 0.004). Among undernourished children with malaria, the risk of late mortality was 1.2% (1/82) in those initially infected with *P. falciparum* and 3.9% (3/77) in those with *P. vivax*. In total 8% (1264/15716) of children had a recurrent episode of malaria within 30 days, of which 36.3% (459/1264) were attributable to *P. falciparum*, 51.1% (646/1264) due to *P. vivax* and 12.7% (161/1264) due to mixed infections. Children with any early recurrence of malaria had an increased risk of late mortality (HR = 1.94 [95%CI: 1.21–3.12]; *p* = 0.006), which was significant following a *P. vivax* recurrence (HR = 2.33 [1.28–4.22]; *p* = 0.005) but not following *P. falciparum* recurrence (HR = 1.34 [0.55–3.28]; *p* = 0.521); Fig. 5, Table 5.

**Table 5:** Risk Factors for late mortality (between 31 and 365 days)

Characteristics	Risk (Events/total cases)	Univariable Analysis Hazard ratio (95% CI)	*p* value
Age Group			
> =1 to <=5	0.6 (61/11005)	Reference	
< 1	1.7 (76/4477)	3.11 (2.22, 4.35)	< 0.001
Gender			
Female	1.0 (70/7346)	Reference	
Male	0.8 (67/8136)	0.86 (0.62, 1.20)	0.38
Ethnicity^a^			
Non Papuan	0.2 (2/1073)	Reference	
Highland	1.1 (131/11545)	6.08 (1.50,24.55)	0.011
Lowland	0.1 (4/2851)	0.74 (0.14, 4.04)	0.728
Inpatient Status			
Outpatient	0.7 (90/12065)	Reference	
Inpatient	1.4 (47/3417)	1.83 (1.28, 2.60)	< 0.001
Anaemia^b^			
Hb > = 7 g/dl	0.8 (49/5918)	Reference	
Hb < 7 g/dl	1.5 (40/2612)	1.83 (1.21, 2.79)	0.004
Malnourished			
Normal	0.9 (132/15290)	Reference	
Undernourished	2.6 (5/192)	2.99 (1.22, 7.30)	0.016
Recurrent malaria within 30 days			
None	0.8 (117/14234)	Reference	
*P. falciparum*	1.1 (5/452)	1.34 (0.55, 3.28)	0.521
*P. vivax*	1.9 (12/635)	2.33 (1.28, 4.22)	0.005
Mixed Infections	1.9 (3/161)	2.34 (0.74, 7.37)	0.145
Species at presentation			
*P. falciparum*	0.8 (47/6106)	Reference	
*P. vivax*	1.0 (72/7378)	1.30 (0.90, 1.88)	0.157
Mixed Infections	0.9 (17/1793)	1.28 (0.73, 2.23)	0.386

^a^ Data missing for 13 children; ^b^ Data on haemoglobin concentrations missing in 6952 children. The low number of late deaths in some subgroups, precluded the presentation of a reliable multivariable model

In the 12,065 children initially managed as outpatients, the only independent predictors of late mortality were being an infant (HR = 3.44 [95%CI: 2.27–5.22]; *p* < 0.001) and malnutrition (HR = 4.64 [95%CI: 1.14–18.85]; *p* = 0.032). Whereas in the 3417 children initially admitted for the management of their malaria, independent predictors of late mortality were being an infant (HR = 2.38 [95%CI: 1.34–4.21]; *p* = 0.003) and having another episode of malaria within 30 days (HR = 3.44 [95%CI: 1.66–7.11]; *p* < 0.001).

The overall incidence of malaria was 2280 [95%CI: 1946-2671] per 1000 py in those who died, compared to 1141 [95%CI: 1124-1158] for those who didn’t die, and similar incidence rates were seen for each initial species at presentation; Table 6.

**Table 6:** Incidence rate of malaria and re-presentation to hospital in those dying and surviving in subsequent 12 months

Recurrence Outcome	Died	Alive
Any malaria	2280 (1946, 2671)	1141 (1124, 1158)
*P. falciparum*	596 (437, 813)	333 (323, 342)
*P. vivax*	1401 (1144, 1715)	634 (621, 647)
Mixed Infections	238 (146, 389)	166 (159, 173)
Inpatient admission	3249 (2845, 3710)	396 (386, 406)
Outpatient presentations	5830 (5280, 6438)	3376 (3347, 3406)

Figures are per 1000 patient years (95% CI)

Excludes 129 patients who died within the first 30 days

### Diagnosis at the time of death

Of the 129 children who died within 30 days of their initial presentation with malaria, 21.7% (28/129) were malnourished, with pneumonia reported in 28.7% (37/129) and diarrhea in 19.4% (25/129). Of those 112 children with documented haemoglobin concentrations, 55 (49.1%) were severely anaemic. Of the 137 children surviving their initial episode of malaria but subsequently dying between 31 days and 1 year, 37 (27.0%) had recurrent malaria at the time of death, 47 (34.3%) were malnourished, 42 (30.7%) had pneumonia and 40 (29.2%) had diarrhea. Of the 113 children with documented haemoglobin concentrations, 47 (41.6%) were severely anaemic at the time of death. There was no significant difference in the risk of these co-morbidities between those presenting with *P. falciparum* and those with *P. vixax*.

## Discussion

The acute morbidity and mortality of *P. vivax* malaria has been documented in a range of endemic settings [3, 4, 8, 22], however the delayed consequences, particularly those associated with recurrent parasitaemia, have not been defined. In the current study the acute and delayed detrimental effects of *P. vivax* on health and survival were assessed in infants and young children in Papua Indonesia, an area co-endemic for *P. vivax* and *P. falciparum*.

At the acute presentation, children with *P. falciparum* infection were 2.3-fold more likely to be admitted to hospital for inpatient care. The 30-day risk of mortality was higher for children with *P. falciparum* than those with *P. vivax* (1.0% vs 0.7%), although this did not reach statistical significance. Conversely, the delayed risk of dying (day 31 to 365) was higher in children initially presenting with *P. vivax* (1.0% vs 0.8%), and by 12 months the cumulative risk of mortality was almost identical between the two species; Fig. 4.

We were unable to identify children fulfilling World Health Organization criteria for severe malaria since the necessary clinical and laboratory criteria were not recorded in our surveillance data (with the exception of haemoglobin). However pharmacy data were documented and allowed us to identify those who were treated with intravenous antimalarial drugs (quinine or artesunate). Intravenous artesunate was introduced for severe malaria due to any species in March 2006, and clinicians have a low threshold for prescribing this in those with clinical concern. Interestingly in patients receiving intravenous therapy, those with *P. vivax* were almost 2-fold more likely to die during their hospital admission compared to those with *P. falciparum* and this difference remained after controlling for confounding factors. Intravenous artesunate is hypothesized to reduce the mortality of *P. falciparum* infection by its rapid reduction in the parasite biomass and prevention of microvascular sequestration and end organ dysfunction [17]. Severe disease in vivax malaria has a lower parasite biomass than that observed in falciparum malaria [23] and its pathophysiology is characterised by severe anaemia, acute inflammation and microvascular dysfunction associated with comorbidities such as malnutrition, pneumonia and diarrhea [7, 8]. In these circumstances a rapid reduction of parasite biomass may have less impact on the clinical outcome.

In the current study, almost half of the children treated for malaria re-presented within 12 months with a further episode of malaria, some children experiencing up to 11 recurrences. The overall incidence of malaria after an initial episode was 1146 per 1000 py. The ability of *P. vivax* to form dormant liver stages, results in the parasite relapsing weeks to months following the primary infection and this was reflected by those presenting with *P. vivax* being at significantly greater risk of any recurrence and multiple recurrences compared to those presenting with *P. falciparum.*

Early parasite recurrence following discharge increased the risk of late mortality by almost 2-fold, and this effect was apparent throughout the 12 months of follow-up (Fig. 5), suggesting that early parasite recurrence may exacerbate or reflect the presence of other risk factors for death. Patients with early recurrent parasitaemia may also be at greater risk for subsequent malaria infections. Indeed, those patients who died within a year had a 2-fold higher incidence of malaria compared to those who survived.

Pro-inflammatory cytokines associated with acute parasitaemia can induce anorexia and increase catabolism, and this is likely to contribute to malnutrition which has been associated with recurrent malaria [24–26]. In the Pacific, children with *P. vivax* infections have been shown to be at higher risk of malnutrition compared to those with *P. falciparum* infections [27].

The chronic and relapsing nature of *P. vivax* results in repeated episodes of haemolysis and dyserythropoeisis [6, 28]. When parasite recurrence occurs prior to haematological recovery, there is a cumulative risk of severe anaemia which, when coupled with malnutrition and sepsis, can result in a fatal outcome [7, 29]. In the current analysis, severe anaemia (Hb < 7 g/dl) was associated with a 2-fold increased risk of delayed mortality and malnutrition with a 3-fold increased risk.

The under-five mortality rate in at the RSMM hospital, during the study period, was 61 per 1000 live births, with 72% of deaths occurring within the first 7 days of life (unpublished data). Hence in babies surviving the neonatal period, the overall risk of dying was approximately 0.3% per year. In the current analysis the corresponding risk of mortality in young children presenting with *P. vivax* malaria was conservatively estimated at 1%, three fold higher than expected. Our study suggests that recurrent episodes of malaria may have contributed both directly and indirectly to this high mortality rate. At the time of death, only 42% of children were associated with another episode of malaria, whereas, 28% were malnourished, 45.3% had severe anaemia and 55% presented with infective comorbidities such as pneumonia or diarrhea. Whilst we are unable to discern the proportion of deaths directly attributable to recurrent episodes of malaria, our findings suggest that the management of acute malaria must include both acute treatment of peripheral parasitaemia, malnutrition and severe anaemia, as well as interventions to prevent subsequent malaria recurrence, such as insecticide treated bednets, prophylaxis or radical cure. The latter requires administration of a combination of a blood schizontocidal drug with a hypnozoitocidal agent. The only currently available drug that targets the dormant liver stage is primaquine, an 8-aminoquinoline compound, which can cause severe heamolysis in G6PD deficient individuals. Concerns over its potential toxicity have limited its use in young children. The World Health Organization recommends that in areas where G6PD testing is unavailable, the use of primaquine should be based on careful assessment of risk and benefit of the treatment [30]. Our findings highlight the significant risks of not providing effective antirelapse therapy.

This study has several important limitations. Firstly, the medical records collected included limited clinical data and relied upon the diagnosis made by the attending physician, coded according to the International Classifications of the Disease 10 (ICD-10). The diagnosis of severe malaria was not captured, however, patients with severe malaria would be more likely to be admitted and be treated with intravenous antimalarials. We assumed that all deaths from severe malaria would have occurred within 31 days. In a previous study at the same location, we showed the important contribution of sepsis and co-morbidities to malaria attributable mortality [7]. Since our current analysis did not quantify the effects of recurrent episodes of non-malarial illness to mortality, we cannot infer the fraction of late mortality attributable to recurrent malaria compared with other comorbidities. However we speculate that recurrent episodes of malaria result in a cumulative risk of anaemia and malnutrition that increases the vulnerability of infants to severe manifestations of other concomitant infections common in early childhood. In support of this, the incidence of malaria was much greater in children who died than those who survived (Table 6), yet when death occurred after 30 days only 27% of cases were associated with recurrent malaria.

This study was limited to patients attending RSMM for healthcare who were mainly from one of the 7 Papuan ethnic groups, are entitled to free health services. Since we did not gather data from patients who represented at other healthcare facilities, a proportion of patients who died in the community or visited other health facilities for malaria treatment would have been missed. The primary aim of this analysis was to determine the comparative risks of recurrent malaria and death between patients initially presenting with *P. falciparum* versus those presenting with *P. vivax*. Previous community surveys have shown that patients with malaria due to either *P. falciparum* or *P. vivax* have a similar tendency to seek medical attention at the same location and in this region, a high proportion of deaths in young children occur at RSMM hospital [12, 13]. We therefore assumed that the attrition bias in detecting the clinical endpoints of the study were similar between species. Hence our comparative analysis remains valid and indeed our findings represent conservative estimates of the risk of recurrence and early and late mortality. In view of the co-linearity between the year of observation and malaria treatment protocol, this study could not compare the effect of treatment on mortality. Hence our analysis represents comparative mortality for the current standard of care at presentation.

Lastly, our estimates of the comparative crude incidence of malaria in those who died versus those who survived are almost certainly biased by the development of immunity and also by differences in age. Children who die are most likely to do so before the development of effective immunity and therefore will have a greater chance of clinical malaria episodes leading up to death than those who survived. Those who died were on average younger than those who survived and it is well recognized that very young children have the highest incidence of malaria (particularly due to *P. vivax*) in Timika.

## Conclusions

Children under 5 years old presenting with malaria due to either *P. vivax* and/or *P. falciparum* have a significant risk of representation with malaria, admission to hospital and both acute and delayed mortality. Recurrent malaria, malnutrition and anaemia were associated with an increased risk of delayed mortality. In the context of malaria elimination in Indonesia, our study suggests that focused efforts are warranted to ensure that early detection and prompt, effective treatment aimed at both eradication of the acute infection and prevention of subsequent recurrence should be delivered in this high risk population.

## Abbreviations

ACT: Artemisinin Combination Therapy
CI: Confidence Interval
DHP: Dihydroartemisinin piperaquine
G6PD: Glucose-6*-phosphate dehydrogenase*
HR: Hazard Ratio
ICD-10: International Classifications of the Disease – 10
PQ: Primaquine
py: person-years
RSMM: Rumah Sakit Mitra Masyarakat (Mitra Masyarakat Hospital)
SD: Standard deviation
